# Mapping the evolution of 'food deserts' in a Canadian city: Supermarket accessibility in London, Ontario, 1961–2005

**DOI:** 10.1186/1476-072X-7-16

**Published:** 2008-04-18

**Authors:** Kristian Larsen, Jason Gilliland

**Affiliations:** 1 The University of Western Ontario, London, ON, N6A 5C2, Canada

## Abstract

**Background:**

A growing body of research suggests that the suburbanization of food retailers in North America and the United Kingdom in recent decades has contributed to the emergence of urban 'food deserts', or disadvantaged areas of cities with relatively poor access to healthy and affordable food. This paper explores the evolution of food deserts in a mid-sized Canadian city (London, Ontario) by using a geographic information system (GIS) to map the precise locations of supermarkets in 1961 and 2005; multiple techniques of network analysis were used to assess changing levels of supermarket access in relation to neighbourhood location, socioeconomic characteristics, and access to public transit.

**Results:**

The findings indicate that residents of inner-city neighbourhoods of low socioeconomic status have the poorest access to supermarkets. Furthermore, spatial inequalities in access to supermarkets have increased over time, particularly in the inner-city neighbourhoods of Central and East London, where distinct urban food deserts now exist.

**Conclusion:**

Contrary to recent findings in larger Canadian cities, we conclude that urban food deserts exist in London, Ontario. Policies aimed at improving public health must also recognize the spatial, as well as socioeconomic, inequities with respect to access to healthy and affordable food. Additional research is necessary to better understand how supermarket access influences dietary behaviours and related health outcomes.

## Background

A growing body of research suggests that the suburbanization of food retailers in North America and the United Kingdom in recent decades has contributed to the emergence of urban 'food deserts', that is, socially-distressed neighbourhoods with relatively low average household incomes and poor access to healthy food [1]. While more and more large-format supermarkets are erected on suburban lands, smaller grocers in older central-city neighbourhoods seem to be rapidly disappearing, leaving potential food deserts in their wake. This paper explores the historical and geographical evolution of supermarket access in a mid-sized Canadian city: London, Ontario, 1961–2005.

Why examine access to supermarkets? A healthy diet can reduce the risk of many chronic diseases [2]. The majority of these health problems can be linked to a diet with low fruit and vegetable consumption [3,4] and eating large amounts of sugary or high fat foods [5]. National surveys indicate that most Canadians shop for food at a local supermarket, where the widest variety of products can be found at the most competitive prices [6]. While supermarkets also carry unhealthy foods (e.g., chips, soft drinks, and processed foods), these items are more readily available at neighbourhood convenience stores, which are less likely to offer items supportive of a healthy diet. What happens to residents when the only supermarket in a neighbourhood closes? For certain disadvantaged populations who do not have access to a private vehicle, residing in a food desert may have detrimental effects on overall health and quality of life [7,8].

Environmental inequity occurs when a locality has disproportionately many undesirable characteristics and few desirable characteristics [9]. While environmental equity research is almost always focused on the 'undesirables', that is, identifying demographic disparities in exposure to environmental hazards such as air pollution, toxic waste and other LULUs (locally unwanted land uses), our concern in this paper is the equitable distribution of a particularly 'desirable' sub-category of land use which offers easy access to an array of food and household items at competitive prices: the retail supermarket.

Our primary objective in this paper is to use network-based GIS accessibility measures to determine the extent to which food deserts exist in London. In order to meet this objective, the paper will provide answers to three fundamental research questions.

1. *Do systematic **spatial inequalities** in access to supermarkets exist?*

2. *Have spatial inequalities in access to supermarkets increased or decreased ****over time***?

3. *Do systematic ****socioeconomic inequalities ****in access to supermarkets exist?*

The scholarly contribution of this paper is twofold: first, for spatial equity research, it offers network-based GIS methods which consider two low-cost travel modes (walking and public transit) for determining accessibility; and second, it gives critical contemporary and historical insights into an urban health issue of increasing present-day interest: the accessibility of healthy and affordable food for disadvantaged populations.

### Urban Development, Grocery Retailing Trends and Access to Healthy Food

Grocery retailing practices in North American cities have undergone many changes over the past century which have largely been driven by prevailing patterns of urban development [10]. The introduction of the automobile has perhaps had the largest impact on shaping cities over the last century, allowing people to move about much further and more freely, fuelling the rapid postwar suburbanization of the population and the deliverers of the goods and services which they consume [11]. Investment in highway infrastructure, particularly in the period immediately following WWII, cemented the dominance of the automobile as the preferred mode of transit. This form of privatized mobility, however, was not democratically distributed; only those with sufficient wealth could afford the luxury of auto-mobility [12].

The automobile provided the opportunity for many residents to move to the suburbs, but it was a cultural ideal that stimulated the move to the suburbs from the inner-city. North American society views cities as unhealthy places to live [13], with poor sanitary conditions, high pollution, unsafe and cramped living spaces. The suburbs were seen as the binary opposite providing healthier opportunities for its inhabitants [13]. At first, retailers and offices remained in the city while residents moved to the suburbs; however, by the 1970's many businesses and retailers (including grocery stores) were moving closer to their predominately suburban customer base [11].

Food retailing, like most other forms of retailing has seen an increase in both store size and the implementation of chains. In the mid 19^th ^century food was typically obtained through small independently owned markets which were an integral part of residential neighbourhoods. By the turn of the 20^th ^century food retailers had begun to organize into chains, most notably the 'The Great Atlantic and Pacific Tea Company' (A&P)[14]. Chaining allowed retailers to lower operating costs, increase profits [14] and use sophisticated location analysis to provide the most rational spatial arrangement of stores for maximal profits [15]. These resources were not available to independent food stores as they could not afford the associated costs. Chaining was followed by the formation of supermarkets in the 1930's, which had a larger selling area and offered a greater selection of goods [14]. Thus, with stores growing larger in size and investment in location analysis, it was inevitable that the overall number of grocery stores per capita would decrease, and retailers would likely follow their wealthier customers to the suburbs.

Most recent to emerge in the urban food retailing landscape is the giant grocery 'superstore', which can be defined as: a single level store with at least 25 000 square feet of sales area, which sells an array of different foods and household items, and includes a very large parking lot [14]. The establishment of giant superstores on suburban lands meets the physical needs for store development as there is more land available for parking, it is easier for trucks to load and unload, these lands are usually more accessible to highways, and they allow for the development of much larger stores [10]. While superstores are often cheaper to construct in suburban neighbourhoods, they still have created new challenges for planners and engineers around traffic, parking, noise attenuation, public transit and other related issues [16].

The relocation of supermarkets has been associated with urban development and the physical need for more space, but economic interests were of course the driving force and the fact that major chain stores, from research and experience, typically know where to locate to maximize profit. The location of supermarkets has always been within close proximity to customers with money [17], while older neighbourhoods have filtered to lower socioeconomic status, the suburbs have typically become more affluent areas; as wealthy residents left the city in favour of suburban living, the supermarkets followed [10]. The effect of the economies of scale parallels the movement of much larger stores as they allow operations to become more efficient [14]. Superstores were able to increase profit as they had lower operating costs, a greater product range, and larger catchment-areas, creating spatial monopolies within their region [14]. Larger stores also meet the social needs of its residents; they have the shelf space to carry many multicultural food items demanded by today's postmodern society and allow for 'one-stop' shopping, as people can find numerous household items along with groceries under one roof, saving valuable time [14].

The rise of the suburban superstore and abandonment of smaller inner-city supermarkets has also presented challenges for planners and public health policy-makers due to the uneven distribution of healthy, affordable food opportunities [7,18-24]. Several U.S. studies have discovered food deserts in older urban neighbourhoods of low income and high Hispanic or African-American populations [25-27]. On the other hand, recent studies of Canadian cities suggest that urban food deserts are not a problem in Canada. A study of the city of Edmonton, Alberta, found that low-income neighbourhoods near the city centre actually had the best supermarket access [28]. Furthermore, a recent article in the *International Journal of Health Geographics *by Apparicio and colleagues found that food deserts are "missing" in Montreal and access to healthy food is not a major issue for low income urban residents [29]. Meanwhile, results of studies of food access in the United Kingdom are mixed, suggesting that no clear relationship exists between supermarket access and variables such as location, income, or race in the cities of the U.K. [23,30,31].

The current literature is inconclusive as to whether easier access to healthy/unhealthy foods influences one's overall diet; however, it is widely acknowledged that a healthy diet can reduce the risk of many chronic diseases, including type 2 diabetes, heart disease and certain types of cancer [2]. The majority of these health issues are associated with a diet low in fruit and vegetable consumption [3,4] and high in both fats and sugar [5]. Two American studies have found that African-American residents were more likely to eat a healthy diet when they had access to a local supermarket compared to smaller grocery retailers [27,32].

Research has also determined that when living in a food desert, residents must pay higher prices for groceries at small food shops and convenience stores [33-40]. Studies in London, Ontario and the nearby Waterloo Region have found that residents will have to pay an average of 1.6 times more for identical food items purchased at area convenience stores versus area supermarkets [41,42]. Furthermore, certain disadvantaged populations, including the elderly, disabled, unemployed, and lone-parent households, would be particularly vulnerable to the limited options in a food desert, as a function of low income and restricted mobility [7,43,44].

In North American cities today, the reality is that most new grocery superstores are found, along with other 'big box' outlets, in expansive retail centers which are almost always built in excess of a 500 metre walk of residential land uses, which essentially makes them accessible only to consumers with automobiles [39]. While planners have devoted a great deal of attention in recent years to identifying the "walkability" of different built environments in relation to physical activity, and by extension obesity [45-50], critical dimensions of food environments have not received the same degree of attention [51,52].

## Methods

### Measuring Supermarket Accessibility

Supermarket accessibility is measured in relation to two low-cost modes of intra-urban travel: walking and public transit. Although previous U.S. studies have demonstrated that even people living in poor neighbourhoods often drive to get groceries [18], we argue that in Canadian cities such as London, Ontario, many people, especially those in lower-income neighbourhoods, must walk or use transit to get groceries. Although the Canadian Census does not include data on automobile ownership, a recent study indicated that only 54% of households in the four most socially-distressed neighbourhoods in London had access to a private automobile [53].

### Data Collection

A spatially-referenced database was created to explore certain historical and geographical dimensions of food deserts for every supermarket in the city in 2005 (n = 28) and 1961 (n = 25). Supermarket addresses were gathered from local business directories, [54,55] and verified using several additional sources: telephone directories (yellow pages); company websites; phone calls to retailers; inspection of air photos, maps, plans; and site visits. The data was geocoded within GIS (ArcGIS 9.2, ESRI) and manually reviewed to ensure extremely high precision: to the front door of each supermarket.

The year 1961 was selected for the beginning of the study, as it was the oldest Census year for which local business directories clearly identified locations of businesses by type [54]. More importantly, four decades was an ideal period of time to observe changes due to the process of suburbanization. The City of London undertook major annexations at the urban/rural fringe during the mid-1960s, which was followed by large-scale suburbanization of businesses and residences. The year 2005 was the most current data available at the time of this study [55].

### Measuring Accessibility in GIS

GIS-based techniques were used to determine the accessibility of supermarkets, or the relative ease with which Londoners can reach a supermarket on foot or public transit. In order to detect potential spatial variations in accessibility, the city was classified into three areas: urban, suburban, and rural. For this study of urban food deserts, rural areas were excluded. To distinguish which parts of London were urban, suburban, and rural we scrutinized phases of historical urban development, particularly how the official city limits expanded over time. Following the practice of local planners, we considered the area within the limits of the City of London as of 1959 to be urban, the area annexed to the City between 1960 and 1992 as suburban, and the lands added since 1993 as rural. Urban and suburban areas were examined in detail at the census tract (CT) level. Census tracts were used as the geographic unit of analysis, as they are commonly-used proxies for 'neighbourhoods' and they are the smallest area unit available that has all the relevant data necessary for the study. In 2001 (the latest census year for which data are available), there were a total of 76 residential CTs within urban and suburban London.

Typical analysis conducted in public health and environmental justice research uses either a 'container' or a 'buffer' approach [56-61]. The container method [61] does not precisely examine proximity; rather, it identifies whether selected features (e.g., hazards) are contained within, or coincide with, a chosen area unit (e.g., county, city, census tract). As such, this method has also been described as 'spatial coincidence' [56] and 'unit-hazard coincidence' [58]. While some previous studies have used a circular 'buffer' to identify all sites located within a pre-determined, straight-line distance (or ring) from origin, the circular buffer technique does not take into consideration how variations in the configuration of the circulation networks along which people actually move can affect levels of access [42,62]. In order to overcome the shortfalls of the classic container or buffer approach, we adopted a network-based approach using the city's circulation system (e.g., streets, footpaths) and public transit routes (bus lines) in calculating the distance one can travel.

### Measuring Accessibility in GIS: Walking

**Figure 1:**
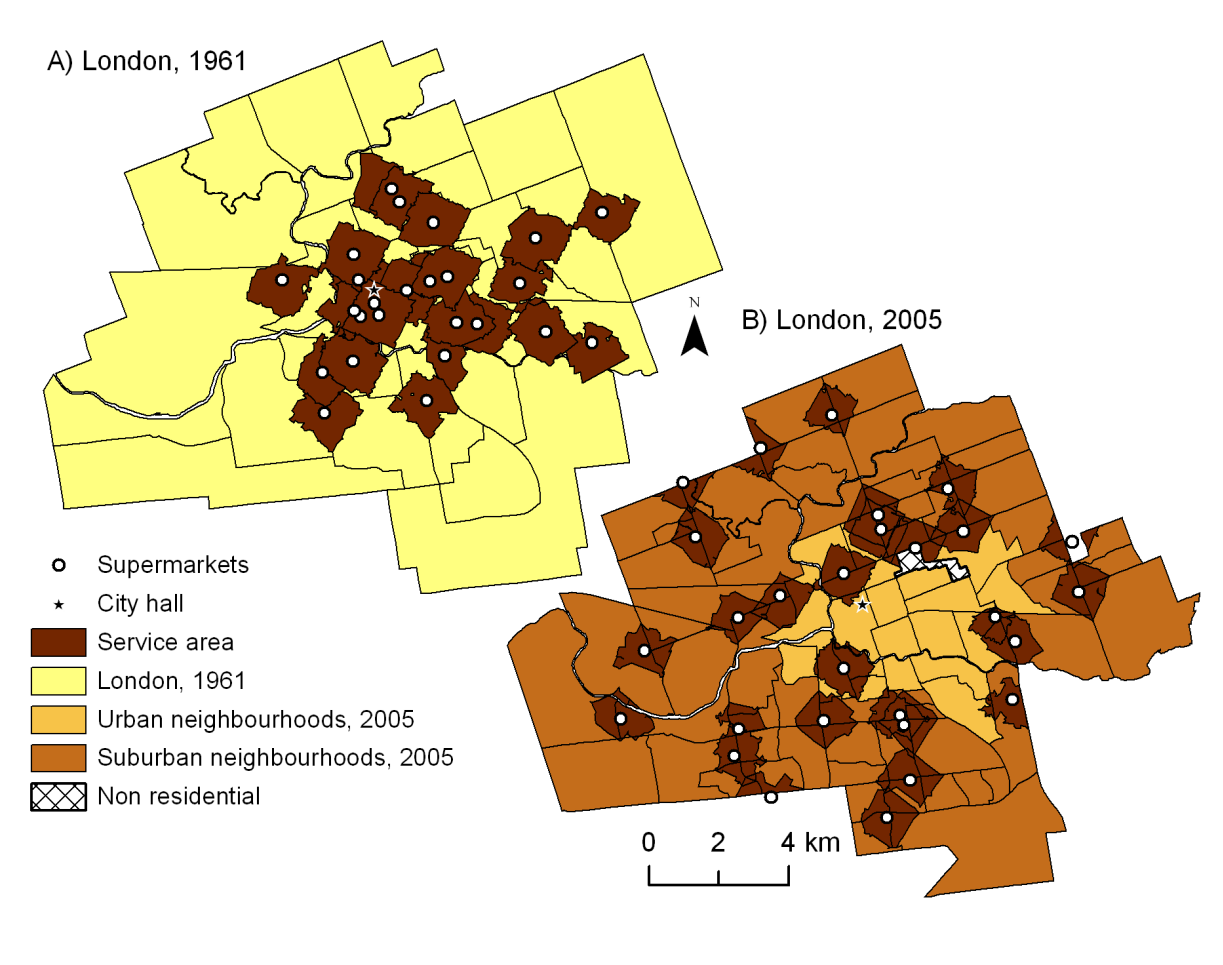
**Accessibility of supermarkets in London (within 1 km), 1961–2005**. *Source: Statictics Canada, 1961, 2001; Vernon's City Directory, 1961, 2005*. Census tracts within the City of London are shaded to indicate urban versus suburban neighbourhoods for 2005 and the City of London for 1961. The locations of supermarkets in London are indicated as points, and are surrounded by 'service areas' that represent areas that are accessible within 1000 metres from a given supermarket along the street network.

Accessibility by foot has typically been measured according to a pre-defined distance from origin (i.e., home). In older food desert studies, a distance of 500 metres was commonly used to assess accessibility by walking [63-65], whereas more recently, two Canadian studies use a distance of 1000 metres [28,29] (or a 10–15 minute walk) to represent accessibility. We originally used both 500 metre and 1000 metre distances in our analyses [42], but for sake of brevity and comparison with other Canadian studies, we will report the results for 1000 metre distances only (additional tables available upon request). Network analysis was employed to create a 'service area' of 1000 metres around each supermarket using the London 2005 street network file (obtained from the City of London Planning Department) within ArcGIS 9.2 (ESRI). We used the 2005 street file also for the 1961 analysis as a careful comparison against the Underwriter's Survey Bureau insurance atlas (1958–62) revealed that the street pattern around the 1961 supermarkets had not changed between 1961 and 2005. Figure 1 illustrates the location of supermarkets in 1961 and 2005 along with the 1000 metre service areas and city boundaries used in this study.

Block level population counts were used to identify the population with supermarket access within each service area. Blocks are the smallest geographic unit for which Statistics Canada releases population counts, and are very useful units for precise spatial analysis. (Unfortunately, virtually all other useful data is suppressed at this scale due to privacy policies). The use of block level data is very precise as it accounts for micro-level variations in population density, rather than assuming that the population is evenly distributed. The supermarket service area were divided into sections and assigned to the appropriate CT for analysis at the census tract level. If a block centroid (i.e., the geographic center) fell within a supermarket service area, the population of that block was identified as having supermarket access. This method allows us to determine how many people live within a walkable distance to a supermarket.

### Measuring Accessibility in GIS: Public Transit

**Figure 2:**
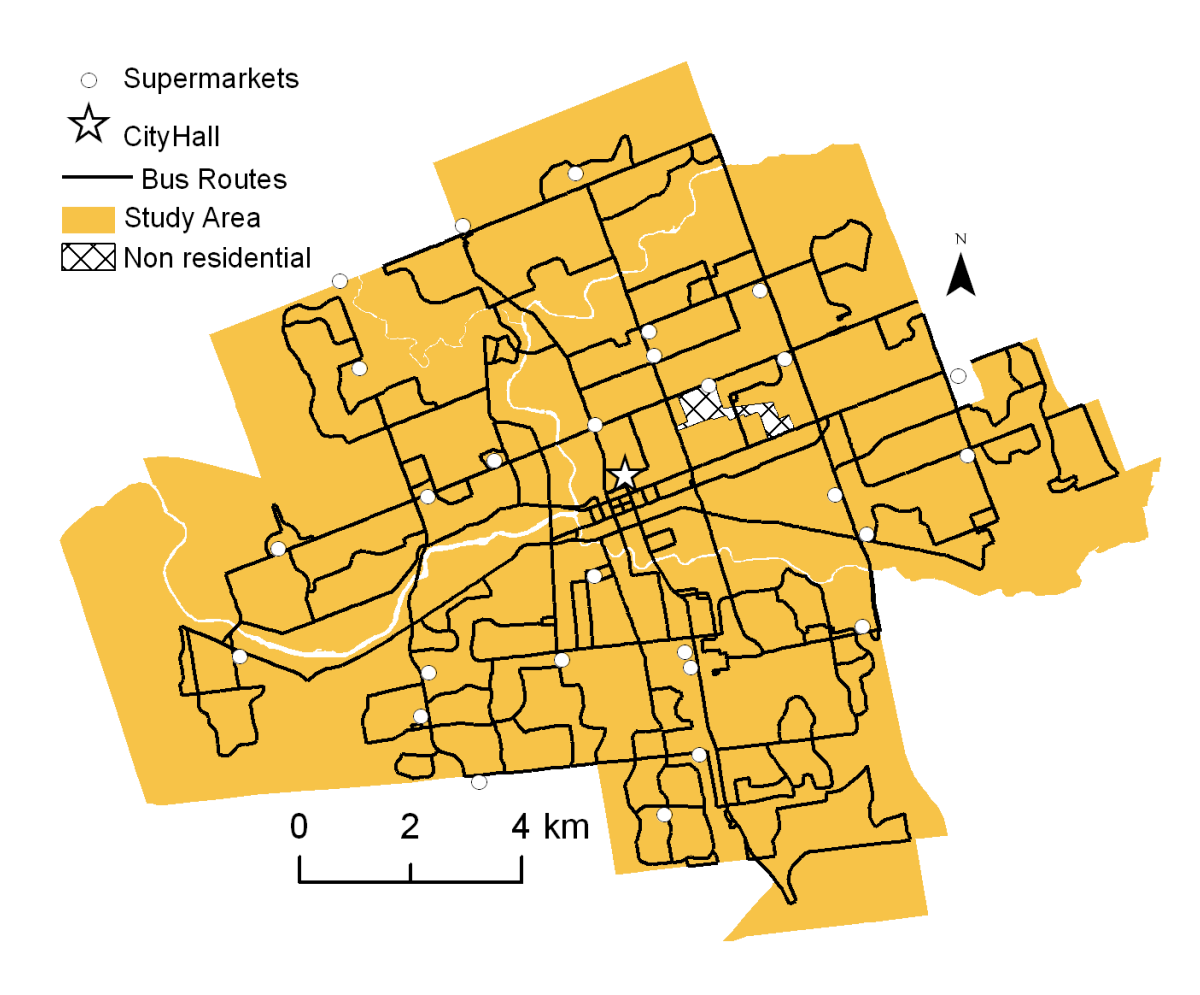
**Location of bus routes and supermarkets in London, 2005**. *Source: Statistics Canada, 2001; Vernon's City Directory, 2005*. Shows the location of the supermarkets in London and all of the bus routes of the London Transit Commission.

Public transit is typically the only option disadvantaged populations have to reach destinations beyond walking distance. We therefore also included data on London's public transit network in our analysis to determine accessibility by city bus. Bus access was determined by using a 10-minute bus ride without transfers, combined with a 500-metre walk at the beginning and/or end of the bus trip. Figure 2 displays the location of bus routes and supermarkets within the city of London. As expected most bus routes permeate inner-city neighbourhoods.

Bus schedules and route data of the London Transit Commission (LTC) were used to determine that a 10-minute bus ride covers a trip of 3 kilometres. Network Analyst was used to select and map individual bus routes that are located within 500 metres and extend up to 3 kilometres from each supermarket; a 500-metre network service area was created around each identified bus route to determine areas with public transit access to supermarkets. Public transit is an option often ignored in previous food desert studies and allows us to more accurately represent supermarket access for London residents.

### Distance to Closest Supermarket

The distance to the closest supermarket was computed using the street network file and the Network Analyst extension within ArcGIS 9.2 to determine proximity or the minimum distance residents must walk to the closest supermarket. This measure calculates the shortest path along the street network from every block centroid (within the urbanized area) to the closest supermarket. The data were then aggregated to the census tract level for further analysis and to allow comparison with socioeconomic characteristics of neighbourhoods, in the same manner as previous Canadian studies (equation 1) [28,29].

(1) mean distance to nearest supermarket (census tract) = ∑*tp *(min|*dps*|)/∑*tp*

*p *= block population

*s *= supermarket

*tp *= total block population within entire census tract

*dps *= distance between block centroid and supermarket

### Number of Supermarkets within 1000 metres

Network Analyst was also employed to calculate the number of supermarkets within 1000-metres of each block centroid. This allows researchers to determine diversity or how many supermarkets residents have access to by foot. Once again the data were aggregated to the census tract level (equation 2) in order to compare with other socioeconomic datasets.

(2) mean number of supermarkets within 1000 metres (census tract) = (∑*tp *∑*sc*)/∑*tp*

*p *= block population

*c *= census tract

*s *= supermarkets

*sc *= number of supermarkets within 1000 metres of block centroid

*tp *= total block population within entire census tract

### Assessing Neighbourhood Socioeconomic Status

To test hypotheses regarding neighbourhood socioeconomic characteristics and supermarket accessibility we first devised a strategy for identifying the relative socioeconomic status (SES) of each CT. Following previous studies of 'disadvantaged', 'deprived' or 'underclass' neighbourhoods [57,60,66-68], census tracts were characterized by a composite index of socioeconomic distress comprised of four variables drawn from the 2001 Canadian census: low educational attainment (proportion of individuals that have not graduated from high school), lone parenthood (proportion of lone parent families versus the total number of families), unemployment (unemployment rate), and incidence of low income (proportion of households that fall below the low income cut-off according to Statistics Canada). Each individual variable was considered on a one-by-one basis, then together in a composite index. Although each of the four variables is positively correlated with the other three (Table 1), we incorporated them all into one composite index in order to highlight the neighbourhoods with multiple indicators of distress [57]. Following the method used by Gilliland and Ross [57], we first calculated the z-scores (based upon un-weighted mean and standard deviation of the indicators) of each CT for each of the four indicators, and then summed them to get a total distress index score for each CT. The census tracts were then classified into three categories (low distress, moderate distress, and high distress) for comparison and analysis.

**Table 1:** Correlation between Level of Neighbourhood Distress and Supermarket Access

		(a)	(b)	(c)	(d)	(e)	(f)	(g)	(h)	(i)
(a)	Lone-parent families (%)	1								
(b)	Incidence of low income (%)	0.802**	1							
(c)	Low educational attainment (%)	0.626**	0.587**	1						
(d)	Unemployment rate (%)	0.464**	0.522**	0.541**	1					
(e)	Distress level	0.787**	0.724**	0.759**	0.689**	1				
(f)	Nearest supermarket (m)	0.071	0.153	0.051	-0.003	0.045	1			
(g)	Number of supermarkets within 1000 m	0.061	0.009	0.020	-0.030	-0.063	-0.685**	1		
(h)	% Population with access 1000 m network	-0.079	-0.201	-0.014	-0.054	-0.101	-0.828**	0.746**	1	
(i)	% Population with bus access	0.238*	0.034	-0.003	0.090	0.104	-0.450**	0.278*	0.420**	1

** Correlation is significant at the 0.01 level (2-tailed).

* Correlation is significant at the 0.05 level (2-tailed).

## Results

### Do Systematic Spatial Inequalities in Access to Supermarkets Exist?

Spatial inequalities in access to supermarkets were explored in urban versus suburban London; the location of all 28 supermarkets with 1000-metre network-based service areas is displayed in Figure 1A. Initial analysis indicates that several regions of the city appear to have limited access to supermarkets, and therefore may be considered 'food deserts' (Figure 1) [69,70]. Several large areas of the suburbs were not within walking distance of a supermarket and there was also a large food desert covering the city centre and areas immediately east, in neighbourhoods known locally as 'Central London' and 'East London'.

Table 2 displays the average proportion of neighbourhood population with supermarket access based on walking and transit access. In both measures the urban areas had better access than the suburban neighbourhoods.

**Table 2:** Proportion of census tract population with supermarket access

Districts	% population with access 1000 m	% population with bus access*
Urban	35.1%	86.5%
Suburban	27.1%	80.8%
London	29.1%	82.4%

* Bus access uses a 10 minute bus ride and 500 metre walk

### Have Spatial Inequalities in Access to Supermarkets Changed Over Time?

The city of London has gone through dramatic changes over the past four decades and the city's population has nearly doubled (population 1961: 169 569; population 2005: 325 045) [69,70]. To establish whether spatial inequalities in access to supermarkets have changed over time, we examined London's supermarket locations in 1961 and compared the population served by each of the major grocery retailers in 1961 (n = 25) and 2005 (n = 28). Figure 1 illustrates changes in supermarket location and access from 1961 to 2005. Analysis indicates that supermarket access has diminished over time, as the average proportion of census tract population with easy supermarket access in 1961 (45.2%) was more than twice the level for 2005 (18.3%) [69,70].

**Figure 3:**
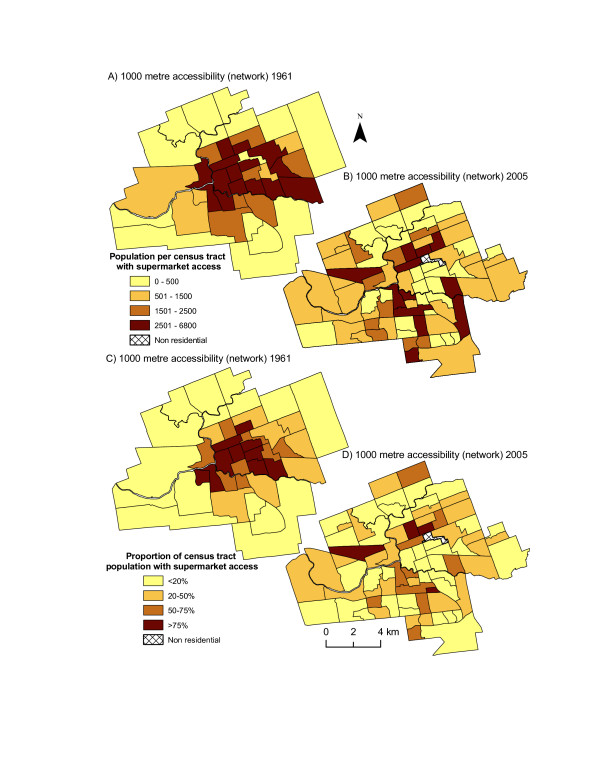
**Change in supermarket access, 1961–2005**. *Source: Statistics Canada,1961,2001; Vernon's City Directory,1961,2005*. The estimated population in each census tract that had access to a supermarket within 1000 metres in 1961 (A) and 2005 (B). Supermarket access was estimated using network-based accessibility measures. The proportion of population with access was also examined for 1961 (C) and 2005 (D).

The number of people in each CT with easy supermarket access in 1961 and 2005 was measured using 1000-metre network-based service areas, and a comparison of the maps in Figure 3 reveals that people in the central core of the city in 1961 were much better served than they are today. In 1961, the majority of CTs in the core of the city had greater than 2500 residents within easy walking distance of a supermarket (Figure 3A); whereas in 2005, only a third of urban CTs had greater that 2500 residents within walking distance, and a third of CTs did not have any residents living within walking distance (Figure 3B). When the proportion of residents with access is considered (Figure 3C; Figure 3D) similar results are discovered. The majority of census tracts in central areas of the city for 1961 had greater than 75% of the population with access to supermarkets, while in 2005 most urban areas displayed that less than 20% of residents had access.

### Do Systematic Socioeconomic Inequalities in Access to Supermarkets Exist?

To explore socioeconomic inequalities in relation to supermarket access we first characterized each neighbourhood by a composite index of socioeconomic distress. The results reported in Table 3 indicate that the most distressed areas of the city had the poorest levels of access to supermarkets by walking, while the least distressed had the best access. When public transit is considered, the most distressed areas of the city had significantly improved access to supermarkets, and the middle class areas had the lowest levels of access, a result of the fact that most bus routes permeate low income urban neighbourhoods.

**Table 3:** Proportion of census tract population with supermarket access according to socioeconomic distress, 2005

Distress	% population with access 1000 m	% population with bus access*
Low	30.3%	81.6%
Moderate	27.5%	79.9%
High	23.8%	86.4%
London	29.1%	82.4%

* Bus access uses a 10 minute bus ride and 500 metre walk

**Figure 4:**
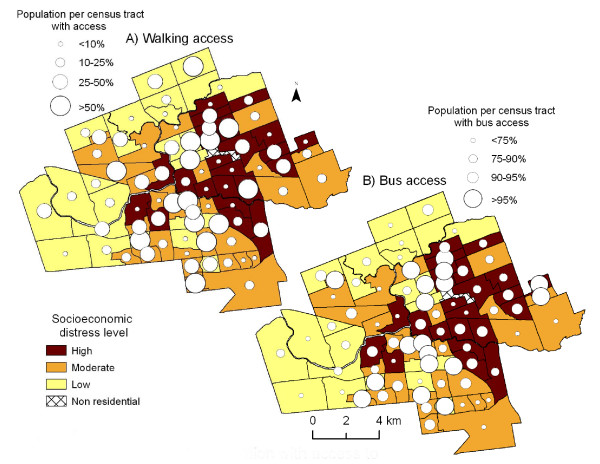
**Proportion of census tract population with access to supermarkets and neighbourhood socioeconomic distress level, 2005**. *Source: Statistics Canada, 2001; Vernon's City Directory, 2005*. Census tracts are shaded according to level of socioeconomic distress, darker shading represents higher scores on a composite index of socioeconomic distress. The graduated circles represent the proportion of census tract with supermarket access, larger circles indicate greater proportions of census tract with access. Figure 4A) represents access by walking, while Figure 4D) demonstrates access via public transit (city bus).

Figure 4A maps the percentage of CT population with supermarket access by foot as graduated circles against a thematic map of CTs shaded according to socioeconomic distress index score. For each CT, larger circles indicate increasing levels of access and darker shading represents higher levels of socioeconomic distress. The results suggest that the most distressed urban CTs have the poorest levels of access to supermarkets. On the other hand, CTs with the lowest levels of socioeconomic distress (suburban areas in the north and west of the city) also have relatively low levels of access to supermarkets; middle class areas appear to be the best served.

If we consider the availability of public transit in our analysis of accessibility, the least distressed neighbourhoods appear to have the poorest access to supermarkets, followed by the most distressed areas. Figure 4B) displays access by public transit in relation to neighbourhood distress level. Figure 4B) again demonstrates that CTs in the highly-distressed East London neighbourhood have the poorest levels of access to supermarkets, while middle-class CTs have the greatest levels of access.

**Figure 5:**
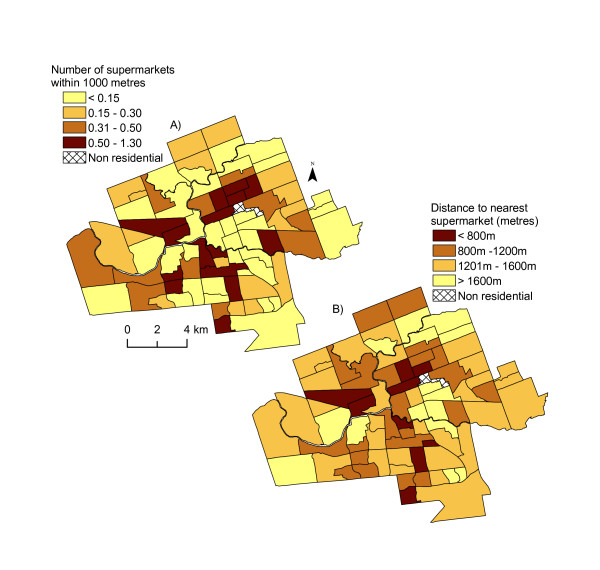
**Supermarket accessibility of census tract in London, 2005**. *Source: Statistics Canada, 2001; Vernon's City Directory, 2005*. Map A) displays the average number of supermarkets within 1000 metres of the block centroid aggregated to the census tract level. Map B) illustrates the mean distance to nearest supermarket from the block centroid, aggregated to census tract.

When we measure access by the number of supermarkets within 1000 metres of each block centroid (aggregated to CT), we do not find significant differences between supermarket access and level of distress, as the figures only varied by 0.07 (Table 4). Figure 5A illustrates the number of supermarkets within 1000 metres, and again the East London region has poorest access to supermarkets.

**Table 4:** Descriptive statistics for supermarket access, 2005

	Number of supermarkets within 1000 m	Nearest supermarket (metres)	% population with access 1000 m	% population with bus access
**Low Distress**				
Mean	0.31	1294	30.3%	81.6%
Median	0.24	1209	25.6%	79.5%
Std. Deviation	0.26	430	23.7%	15.7%
Variance	0.07	184470	5.6%	2.5%
Percentiles				
5	0.00	681	0.0%	44.7%
10	0.01	768	0.0%	59.8%
25	0.11	983	10.4%	73.2%
50	0.24	1209	25.6%	79.5%
75	0.41	1495	49.2%	98.9%
90	0.70	2100	66.7%	99.8%
95	0.96	2204	78.3%	100.0%

**Moderate Distress**				
Mean	0.24	1280	27.5%	79.9%
Median	0.11	1304	19.2%	87.7%
Std. Deviation	0.29	496	27.4%	22.3%
Variance	0.08	246379	7.5%	5.0%
Percentiles				
5	0.00	468	0.0%	24.8%
10	0.00	648	0.0%	41.5%
25	0.00	830	4.6%	67.6%
50	0.11	1304	19.2%	87.7%
75	0.45	1553	45.8%	98.1%
90	0.78	1593	70.8%	99.9%
95	0.80	2447	90.1%	100.0%

**High Distress**				
Mean	0.26	1347	23.8%	86.4%
Median	0.07	1261	23.3%	91.5%
Std. Deviation	0.36	431	24.7%	14.1%
Variance	0.13	186169	6.1%	2.0%
Percentiles				
5	0.00	613	0.0%	45.8%
10	0.00	808	0.0%	71.6%
25	0.00	1042	0.0%	76.1%
50	0.07	1261	23.3%	91.5%
75	0.34	1664	33.5%	96.8%
90	0.95	1976	58.4%	99.2%
95	1.26	2137	89.2%	99.9%

Our measurements of distance to the nearest supermarket suggest that there is no significant difference in supermarket access related to neighbourhood socioeconomic distress level. Residents in areas of the highest socioeconomic distress had the furthest to travel at 1346 metres (Table 4), but this is only about 66 metres further than residents in the best-served areas of moderate distress levels. Figure 5B illustrates the poor access to grocers in East London, where distance to the nearest supermarket is over 1600 metres.

While no significant correlation was found between level of supermarket access and presence of low income (Table 1); the East London neighbourhood is still believed to be a food desert. With all four measures of analysis East London was always in the category representing poorest access to supermarkets.

## Discussion

The suburbanization of grocery stores over the last four decades in London has created a distinct 'food desert' in the central and eastern sections of the urban core. In 1961, the vast majority of grocers in London were located in the inner city and very few were in the suburbs; however, the landscape of grocery retailing is very different today, as supermarkets have left the older, inner-city neighbourhoods such as Central London and East London, and these low-income areas now have the poorest levels of supermarket access by walking. These changes in retailing practices are also driven by transformations in demographics within the city. In 1961, the majority of the population lived in central areas near downtown, while in 2005 these areas near the core have lost population and are now even less dense than a few suburban neighbourhoods. Not surprisingly, the new supermarkets opening in suburban areas have been designed for the car-borne suburban consumer. In fact, the massive parking lots that surround the so-called 'superstores' today have made the present-day grocery shopping trip virtually impossible for pedestrians. The footprint of the typical supermarket in London has increased dramatically from a mere 850 square metres of floor area in 1961 [71] to a whopping 4000 square metres today [72]. These changes in supermarket location and floor area can be attributed to the changing business practices of grocery retailers nationwide.

Since it is somewhat unrealistic to think that everyone should live within walking distance to a supermarket, we also assessed access via public transit. As the majority of the bus lines go through the urban core, we were not surprised to find that most urban census tracts had superior access by transit; however, it is noteworthy that the highly-distressed neighbourhood of East London also had below average level of access by bus. As suggested in previous work [53], we can assume that people in poorer, more distressed, neighbourhoods do not have as high a level of access to private automobiles as people in less distressed, high income areas, and are therefore more likely to walk or rely on public transit to shop for groceries. When walking is not feasible and transit access is not available, residents may use a taxi for transportation, but taxi fare adds a significant cost to the household budget which can be troublesome for persons of low income or fixed incomes. Furthermore, taxi dependency would also necessitate making fewer trips to the supermarket to avoid multiple fares; and, in turn, less frequent trips would be detrimental to the household's ability to take full advantage of time-sensitive sales, to keep fresh produce in the home, and to minimize the total financial outlay for any one shopping trip.

Based on our results with respect to the measures of distance to nearest supermarket and number of supermarkets within 1000 metres, it can be concluded that supermarket accessibility is poor throughout the city of London. Furthermore, according to these measures, supermarket access varied little in relation to level of socioeconomic distress, as each category displayed relatively dismal access. While supermarket access is poor throughout the city of London, the overall findings indicate that distinct food deserts do exist, particularly in the East London neighbourhoods.

Our findings differ in comparison to another Canadian study in this journal by Apparicio and colleagues [29], who found that most areas of the city of Montreal had good access to supermarkets, except for suburban lands with low distress, leading them to claim that food deserts do not exist as a food desert can only be present when there is both poor access and high distress. Although they are both Canadian cities, Montreal and London are very different in terms of population and urban form. Montreal is much larger than London in terms of total population, at 1.8 million versus 350,000 inhabitants, and it also has a much higher population density: 847 versus 185 persons per square kilometre [70]. While food deserts are not always a function of population density, retail businesses such as grocers require a certain customer base (i.e., population density threshold) to be profitable; it is not surprising that London's lower population density is associated with a more dispersed distribution of supermarkets than in higher-density Montreal. Furthermore, London's low-density form makes the city much less 'walkable' than Montreal. This lack of accessibility makes automobile ownership a practical necessity for many routine daily functions such as grocery shopping.

Londoners living in inner-city neighbourhoods of low socioeconomic status have very poor levels of access to supermarkets by foot; indeed, there are no supermarkets in neighbourhoods that locals would associate with 'downtown living'. Ironically, the high income neighbourhoods with the lowest level of socioeconomic distress also had relatively poor supermarket access. This finding is perhaps not surprising, as every census tract in the lowest social distress category was located in a newer suburb of London, where automobiles are a necessity for everyday life. Additionally, the median household income for these suburban areas was typically much higher than other regions of the city: $91,172, versus the city median of $48,026 and $36,583 for East London [70]. In London, as in many North American cities, households of higher income generally reside in larger houses on larger lots, in neighbourhoods of relatively low density, that tend to be zoned exclusively residential; thus, the physical design of the suburban environment dictates that these households will not have good access to supermarkets within walking distance.

### Future Research

While this study makes methodological advances by incorporating GIS-based techniques for evaluating accessibility by foot and public transit in an urban setting, an obvious limitation is the highly empirical nature of the study. The most useful avenue for future research would be to undertake interviews with people who live in food deserts in order to gain a better understanding of the personal, psychological, economic and geographical dimensions of grocery shopping patterns and behaviours. As census data on automobile ownership in London does not currently exist, surveys and interviews are needed to determine levels of vehicle ownership in different neighbourhoods, and how (lack of) access to a personal vehicle influences grocery shopping behaviours.

Conclusions as to whether easy access to supermarkets improves one's diet and overall quality of life have also been mixed [32,73-76]. Residents of a food desert may not have access to a supermarket, but other types of small food retailers such as a local butcher, fruit and vegetable market, baker, or ethnic and speciality food stores may exist. Local accessibility to these kinds of smaller food stores may improve residents' access to healthy foods. Although this type of geographical access may exist, food type, quality, and price should also be examined to help us understand diet and consumer costs of life in a food desert in London, Ontario (and elsewhere).

### Implications for Urban Planners

It can be argued that urban food deserts are merely another product of the social and economic forces underlying the historical evolution of urban form. Basically, new supermarkets are being erected at the suburban fringe and older ones are being shutdown in the inner-city because it makes economic sense for the owners of the supermarket chains, who are following the suburbanization of their customer base. Furthermore, in suburbia, the grocery chains are able to access larger tracts of developable land, often at a much cheaper price than in urban areas, in order to build giant superstores with ample parking. Studies have also found that operating costs in urban locations are typically higher than in suburbs [10]. The bottom line is that major grocery retailers know where to locate a supermarket to maximize profit and are likely to continue the trend of building superstores in the suburbs as long as it is the most profitable solution.

The reintroduction of small supermarkets in Central or East London could have many benefits to not only the citizens currently living in these areas, but it could also help attract more people to the downtown core. London City Councillors have publicly acknowledged the need for a supermarket in the core districts in order to attract and maintain residents and businesses [77]. In order to attract small supermarkets back to the urban core, the municipal government could introduce financial incentives, such as tax breaks or building restoration initiatives. The City could also change zoning or parking regulations in certain areas to encourage grocery chains to consider locations in need. Such incentive programs and regulatory modifications are in line with the mandates of local community organizations *Mainstreet London *and the *Old East Village Business Improvement Area *that are working to bolster economic development in Central and East London respectively [78,79]. In other major cities across Canada (e.g., Toronto, Montreal, Edmonton), supermarkets can still be found in inner-city locations, and new ones are continually being built; but again, these cities are already larger, with higher population densities in their central cores. The bottom-line is that people need supermarkets (the political/popular view), and supermarkets need people (the business view); any municipal strategy for inner-city development or revitalization must recognize the positive correlation between supermarkets and population density. Regardless of the chosen strategy, the City of London should actively encourage supermarket development in areas currently identified as food deserts.

## Conclusion

This study explored issues of environmental equity with respect to the historical evolution of food deserts in London, Ontario. The findings confirmed that some areas of the city currently have significantly better access than others, and the East London neighbourhood is indeed a food desert. Historical analysis, however, indicated that inner-city neighbourhoods such as East London have not always been food deserts. The research suggests how food deserts are the result of structural and geographical changes in the business of grocery retailing over the past few decades, as the majority of supermarkets that existed in the city in the 1960s have since vacated their original urban locations, and the majority of new stores have been built in the suburbs. This study is the first known attempt to present a historical analysis of the spatial patterning of supermarket access in a mid-sized North American city, thereby contributing important new information to our understanding of the evolution of urban food deserts.

A major contribution of this research resulted from the utilization and evaluation of different measures to determine accessibility; the methods outlined here should serve as guidelines for future studies of environmental equity. To further expand the knowledge base on food deserts, access to public transit was assessed to more accurately measure how low-income residents can realistically access supermarkets. This consideration added a very important dimension to this study, as previous studies have failed to examine access via public transit. This failing of previous studies is even more significant considering that most researchers have focussed on larger cities, which tend to have more advanced public transit networks (including subway systems). Our results found that populations in the majority of the urban census tracts had very good access to supermarkets via public transit, but the population in East London still had poorer access by transit, compounding the impacts of the food desert.

While the current retailing practice of building supermarkets only on 'greenfields' at the suburban/rural fringe does not seem to show signs of slowing, this study demonstrates some of the negative consequences of this trend. This practice causes many smaller urban supermarkets to close, and food deserts to form throughout the city. Governments at all levels must evaluate and react to the various consequences associated with the closure of smaller urban supermarkets. Ultimately, the closure of the supermarkets will result in an increased unemployment rate in distressed cores, fewer visitors to surrounding retailers, and potential impacts to the health and well-being of already vulnerable populations.

## Competing interests

The authors declare that they have no competing interests.

## Authors' contributions

KL completed the GIS-based analyses and wrote a draft of the manuscript. JG designed the study, edited and wrote sections of the paper. Both KL and JG performed the analyses. All authors read and approved the final version of the manuscript.
